# Assessing venous thromboembolism risk in lung cancer: a comprehensive evaluation of the khorana score, clinical characteristics, and incidence of thrombosis

**DOI:** 10.3389/fonc.2025.1651192

**Published:** 2026-01-27

**Authors:** Sean C. Dougherty, Rishika Singh, Caleb McKinney, Aditya Singh, Jack Masur, Alia C. Lynch, Ryan D. Gentzler, Wendy Novicoff, Nathan Roberts, Richard D. Hall

**Affiliations:** 1 Division of Hematology/Oncology, Department of Medicine, University of Virginia Cancer Center, Charlottesville, VA, United States; 2 Department of Pharmacy, University of Virginia Health System, Charlottesville, VA, United States; 3 Department of Public Health Sciences and Orthopaedic Surgery, University of Virginia Health System, Charlottesville, VA, United States

**Keywords:** khorana risk score, non-small cell lung cancer, oncogenic driver mutation, small cell lung cancer, venous thromboembolism

## Abstract

**Introduction:**

Venous thromboembolism (VTE) occurrence in patients with lung cancer is associated with significant morbidity and mortality. The Khorana Score (KRS) is a validated risk stratification tool developed to estimate risk of VTE in patients with cancer that incorporates clinical and laboratory parameters to calculate individual risk. However, limitations of the KRS in lung cancer have been reported, and identification of other risk factors associated with increased risk of VTE are needed in this population.

**Methods:**

We retrospectively evaluated 485 patients with advanced non-small cell lung cancer (NSCLC) and 67 patients with small cell lung cancer (SCLC) treated at our institution between March 2015 and April 2023 to assess the performance of the KRS in estimating risk of VTE in multiple clinically distinct subtypes of lung cancer.

**Results:**

The median patient age at diagnosis was 66 years (range, 30-94 years). Patients were predominantly male (51.1%), Caucasian (81%), and had a history of smoking (79.4%). VTE events were observed in 123 patients (22.3%); 51 patients had DVT (41.5%), 50 patients had PE (40.7%), 13 patients had concurrent DVT and PE (7.3%), and other VTE occurred in 9 patients (7.3%). 478 patients (86.6%) had low-risk KRS (score = 0-2) and 74 patients (13.4%) had high-risk KRS (score ≥3). No statistically significant difference in VTE incidence was observed based on baseline KRS. Clinical factors that were noted to be associated with increased risk of VTE were NSCLC histology, Black race, and the presence of an oncogenic driver mutation. No significant differences in overall survival were noted in patients that experienced VTE.

**Discussion:**

This study highlights the limitations of the KRS in predicting risk of development of VTE in patients with NSCLC and SCLC and identifies multiple clinical parameters associated with increased risk of VTE. Development of further risk stratification tools incorporating these clinical factors in addition to those in the KRS should be considered.

## 1 Introduction

Cancer increases risk of venous thromboembolism (VTE) and this risk varies based on specific histologic diagnosis, stage, and type of cancer treatment (1–4). VTE occurrence in patients with cancer can delay treatment, eliminate therapeutic options, increase risk of bleeding from therapeutic anticoagulation, and increase mortality (5–14). Patients with lung cancer, specifically, have increased risk of VTE with a reported incidence as high as 8% in advanced lung cancer patients undergoing treatment with chemotherapy (15, 16). Chew et al. showed that patients with newly diagnosed lung cancer had a 3% 1-year cumulative incidence of VTE and that mortality increased by 2.3 and 1.5 times in patients with non-small cell lung cancer (NSCLC) and small cell lung cancer (SCLC), respectively, after developing VTE (17).

Pharmacologic VTE prophylaxis has been shown to reduce risk of VTE without significantly increasing major bleeding rates in ambulatory patients with cancer (18). However, the most recent American Society of Clinical Oncology guidelines from 2023 suggest that routine VTE prophylaxis for all cancer patients is not supported. Instead, the guidelines specify with moderate strength that only high-risk outpatients, using a cut-off of Khorana Risk Score (KRS) of 2 or higher, should be offered thromboprophylaxis provided there are minimal risk factors for bleeding and a risk-benefit discussion is held with the patient (19). The KRS is a validated risk stratification tool developed to estimate risk of VTE in patients with cancer. The KRS incorporates clinical (body mass index, cancer type) and laboratory (pre-treatment platelet, hemoglobin and white blood cell counts) parameters to calculate individual risk. Following development of their model, Khorana et al. showed that using a cutoff point of ≥3 to define high risk, the KRS had a negative predictive value of 98.5% and a positive predictive value of 6.7% for VTE in cancer patients (20).

In subsequent studies attempting to validate the KRS in lung cancer, limitations have been noted. In 2016, Mansfield et al. published a retrospective study of patients with lung cancer which showed that a high KRS, defined as KRS ≥3, was not associated with increased risk of VTE compared with an intermediate score in patients with both early stage and metastatic lung cancer (21). Despite the lack of association with VTE, however, a high KRS was a predictor of mortality (hazard ratio (HR) 1.7, 95% confidence interval (CI) 1.4 - 2.2). In 2020, van Es et al. showed that while KRS did accurately predict VTE risk in colorectal, pancreatic, gastric, ovarian, breast, brain, and bladder cancer, it did not perform well in assessing VTE risk in lung cancer patients (22).

Given the limitations of the KRS for predicting VTE risk in lung cancer patients, other clinical risk factors have been examined to improve the accuracy of estimating risk of VTE. Among these other risk factors, certain oncogenic driver mutations have been shown to potentially impact VTE risk. Previous studies have identified *ALK* and *ROS1* rearrangements as being associated with increased VTE risk in NSCLC, while *EGFR* and *KRAS* mutations either have no effect or are negatively associated with VTE risk (23–26). Cancer stage, histology subtype, and type of treatment are additional risk factors for VTE that have been reported (16, 27–30). While multiple individual risk factors have been examined in retrospective studies, a comprehensive assessment of these risk factors within a large cohort of lung cancer patients with multiple histologic subtypes, oncogene driver mutations, and receiving a wide variety of treatment modalities for advanced lung cancer is lacking.

The primary objective of this study was to assess the performance of the KRS in estimating risk of VTE in multiple clinically distinct subtypes of lung cancer, including histologic and genomic subtypes. The secondary objectives of this study were to further investigate the impacts of histology subtype, presence of a driver mutation, treatment type, and other clinical parameters on VTE risk in our cohort of patients with advanced stage NSCLC and SCLC.

## 2 Materials and methods

**Figure 1:**
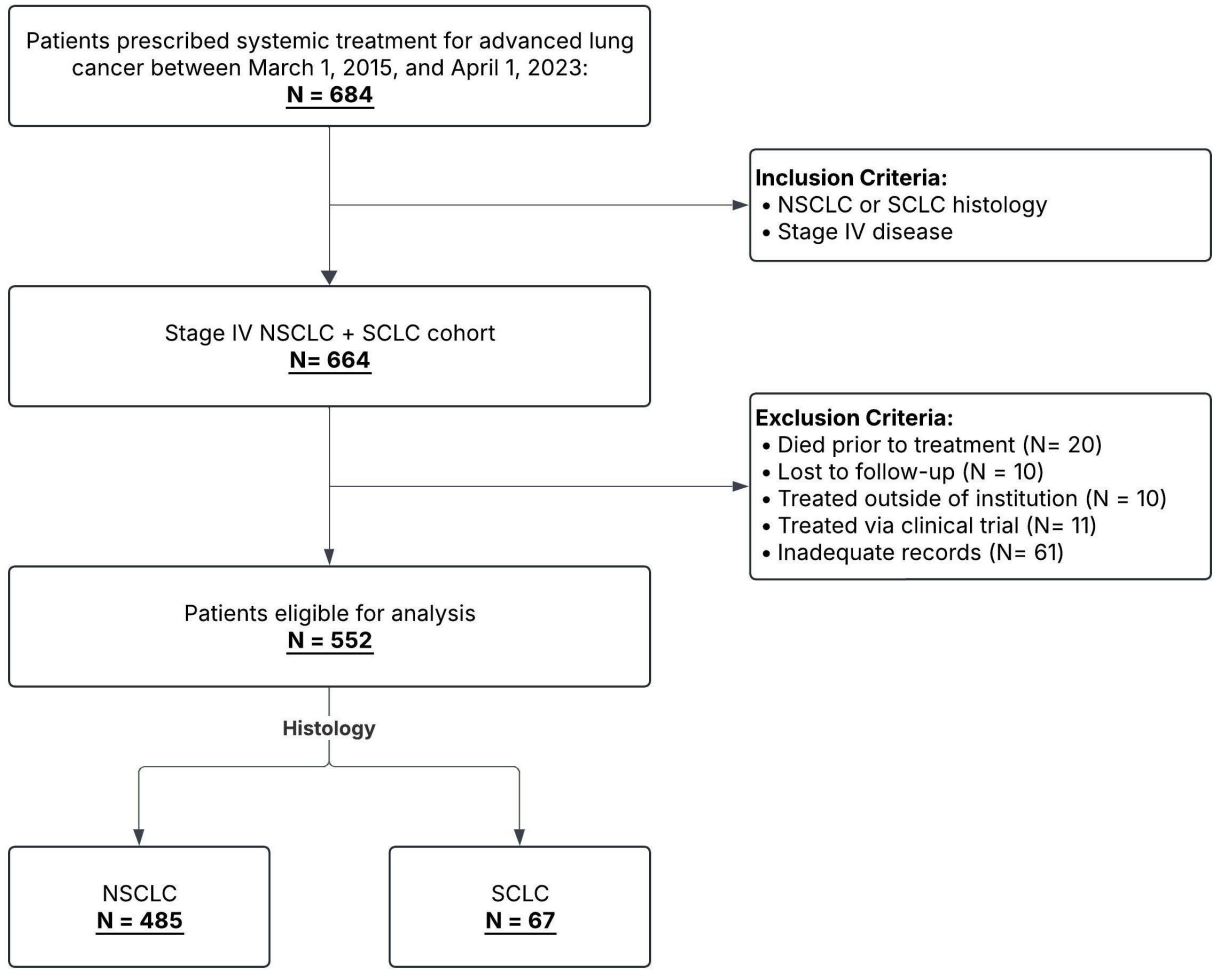
Patient selection flowchart. Flowchart illustrating selection of patients for the study cohort. Inclusion and exclusion criteria are shown; numbers excluded at each step are indicated with the associated reasons for exclusion.

We retrospectively evaluated patients diagnosed with advanced lung cancer treated at our institution between March 2015 and April 2023. Patient demographics, treatment information, and clinical outcomes were obtained by review of the electronic medical record. Our cohort included patients with advanced, stage IV NSCLC and extensive-stage SCLC (ES-SCLC). Patients with NSCLC had either *de novo* metastatic disease or metastatic recurrence of disease; see **Figure 1** for details regarding patient selection. Patients were treated with chemotherapy only (CTX), immunotherapy only (IO), chemotherapy plus immunotherapy (CTX+IO), or tyrosine kinase inhibitor (TKI) monotherapy. Patients that did not receive treatment or were lost to follow-up were excluded. The study was approved by the Institutional Review Board for Health Sciences at the University of Virginia (HSR 18465 and HSR 19083).

A baseline KRS for VTE was calculated for all patients using clinical and laboratory data from the most proximal clinical evaluation prior to initiation of treatment. The score was calculated using the following parameters: primary site of malignancy, pre-treatment platelet count ≥350×109/L, hemoglobin levels <10 g/dL (or use of red blood cell growth factors), pre-treatment leukocyte count >11×109/L, and BMI ≥35 kg/m2. Each component was assigned a score of 1 point, except for site of primary malignancy (high-risk cancers, such as gastric or pancreatic cancer, were assigned a value of 2 points). All patients in this study had a minimum score of 1 given their diagnosis of lung cancer. We classified patients as low risk (score = 1-2) or high risk (score ≥3).

Univariate and multivariate statistics were used to analyze primary and secondary variables. Means, medians, standard deviations, and either 95% confidence intervals (CI) or interquartile ranges (IQR) were computed for all continuous variables, including demographic and treatment variables; proportions and percentages were computed for categorical variables. In univariate analysis, one-way analysis of variance (ANOVA) was used to compare groups for continuous outcome variables, and chi-square tests were used to compare groups on categorical outcome variables. HR with 95% CI were reported for univariate and multivariate models. Landmark analysis was employed to adjust for potential bias in survival outcomes. Kaplan-Meier (KM) curves and Cox proportional hazards regression analyses were used to evaluate overall survival (OS). A p-value less than 0.05 was used to determine statistical significance.

## 3 Results

### 3.1 Patient characteristics

This retrospective cohort study consisted of 552 patients with advanced-stage lung cancer, including 485 patients with NSCLC and 67 patients with SCLC. Patient baseline characteristics and demographics are summarized in **Table 1**. The median patient age at diagnosis was 66 years (range, 30–94 years). Patients were predominantly male (51.1%), Caucasian (81%), and had a history of smoking (79.4%). The median body mass index (BMI) was 25.2 (range 13.4 - 50.9). The median Eastern Cooperative Oncology Group (ECOG) performance status was 1, and median Charlson Comorbidity Index (CCI) was 9 (range, 6-16). 184 patients (33.3%) had brain metastasis present at time of diagnosis. Median follow-up for all patients included in the analysis was 18.6 months (range, 0-138.5 months).

**Table 1:** Patient characteristics.

Total patients, n = 552	
Median age at diagnosis (range) – years	66 (30-94)
Sex	
Male – no. (%)	282 (51.1)
Female – no. (%)	270 (48.9)
Race/Ethnicity – no. (%)	
White/Caucasian	447 (81.0)
Black	74 (13.4)
Asian	9 (1.6)
Hispanic	12 (2.2)
Other	8 (1.4)
ECOG performance status – no. (%)	
0	110 (19.9)
1	305 (55.2)
2 or greater	137 (24.8)
Smoking status – no. (%)	
Former	342 (62.0)
Current	96 (17.4)
Never	111 (20.1)
Median baseline BMI (range)	25.2 (13.4-50.9)
Median Charlson Comorbidity Index (range)	9 (6-16)
Histology	
NSCLC – no. (%)	485 (87.9)
Adenocarcinoma	367 (66.5)
Squamous cell carcinoma	80 (14.5)
Other	32 (5.8)
SCLC – no. (%)	67 (12.1)
Brain metastasis at diagnosis – no. (%)	
Yes	184 (33.3)
No	347 (62.9)
Unknown	21 (3.8)
First-line Treatment – no. (%)	
Chemotherapy	102 (18.5)
Chemotherapy + Immunotherapy	191 (34.6)
Immunotherapy	125 (22.6)
TKI	134 (24.3)

ECOG, Eastern Cooperative Oncology Group performance status; BMI, body mass index; NSCLC, non-small cell lung cancer; SCLC, small cell lung cancer; TKI, tyrosine kinase inhibitor; VTE, venous thromboembolism; DVT, deep vein thrombosis; PE, pulmonary embolism.

The observed oncogenic driver alterations included the following genes: anaplastic lymphoma kinase (ALK), v-raf murine sarcoma viral oncogene homolog B1 (BRAF), epidermal growth factor receptor (EGFR), kirsten rat sarcoma viral oncogene homolog (KRAS), mesenchymal epithelial transition factor receptor (MET), rearranged during transfection (RET), and c-ros oncogene 1 (ROS1). All driver mutations were identified in patients with NSCLC. We observed ALK rearrangements in 20 patients (3.6%), BRAF mutations in 22 patients (4.0%), EGFR mutations in 124 patients (22.5%), KRAS mutations in 100 patients (18.1%), RET rearrangements in 6 patients (1.1%), and ROS1 rearrangements in 8 patients (1.4%).

For first-line treatment regimens, in the entire cohort, 102 patients (18.5%) received CTX only, 191 patients received CTX + IO (34.6%), 125 patients (22.6%) received IO only, and 134 patients (24.3%) received TKI only.

### 3.2 VTE incidence

VTEs included in this analysis were deep vein thrombosis (DVT), pulmonary embolism (PE), superior mesenteric vein thrombosis, splenic vein thrombosis, cerebral venous sinus thrombosis, and internal jugular vein thrombosis. Arterial thrombi were excluded. VTE events other than DVT and PE will be grouped and classified as “Other” VTE from this point onwards.

**Figure 2:**
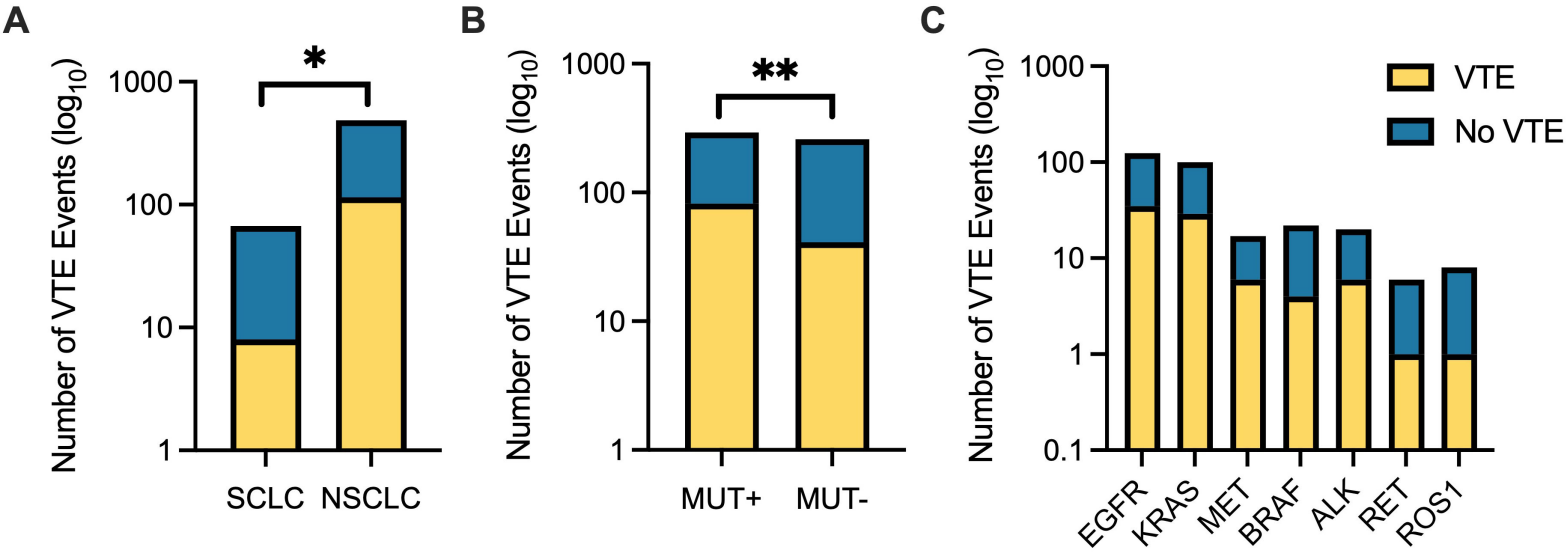
VTE incidence stratified by histology and oncogene driver mutation. **(A)** Patients with non-small cell lung cancer (NSCLC) experienced significantly more VTE events compared to those with small cell lung cancer (SCLC) (p = 0.03). **(B)** NSCLC patients with oncogene driver mutations (MUT+) had significantly higher VTE events than those without identified mutations (MUT−) (p=0.002). **(C)** Distribution of VTE events across specific oncogene driver mutations (*EGFR*, *KRAS*, *MET*, *BRAF*, *ALK*, *RET*, *ROS1*). Data are plotted on a log_10_ scale to illustrate differences in magnitude across subgroups. Yellow bars indicate patients with VTE; blue bars indicate patients without VTE. * = p ≤ 0.05, ** = p ≤ 0.01.

VTE events were observed in 123 patients (22.3%) during the entire follow-up period. Detailed data on VTE are provided in **Figure 2**, **Table 2**, and **Table 3**. 51 patients had DVT (41.5%), 50 patients had PE (40.7%) and 13 had concurrent DVT and PE (7.3%). Other VTE occurred in 8 patients (6.5%); of hese events, 7 were central-line-associated VTE. One patient was diagnosed with PE in addition to an Other VTE. Median time from lung cancer diagnosis to development of VTE was 239 days (IQR 76–913 days); 5 thrombosis-related mortalities were observed. Of patients with VTE, the majority were Caucasian (96 patients, 78.0%); Black race was associated with a statistically significant increase in VTE (Pearson chi-square test, p=0.02), with VTE observed in 24 patients (4.3%). By histology, VTE occurred in 115 patients with NSCLC and 8 patients with SCLC (Pearson chi-square test, p=0.03). No statistically significant difference in VTE incidence was observed by treatment type (Pearson chi-square test, p=0.13).

**Table 2:** Types and timing of VTEs.

Total VTE events, n = 123	
Type of VTE – no. (%)	
DVT	51 (41.5)
PE	50 (40.7)
DVT + PE	13 (10.5)
Other	9 (7.3)
Median time to VTE:	
Histology (range) – days	
NSCLC	264 (58-1108)
SCLC	162 (39-292)
First-line Treatment (range) – days	
Chemotherapy	301 (86-1005)
Chemotherapy + Immunotherapy	138 (74-532)
Immunotherapy	203 (58-924)
TKI	443 (114-1108)
All VTE+ Patients (range) – days	239 (58-1108)

DVT, deep vein thrombosis; PE, pulmonary embolism; NSCLC, non-small cell lung cancer; SCLC, small cell lung cancer; VTE, venous thromboembolism; TKI, tyrosine kinase inhibitor.

**Table 3:** VTE incidence by patient characteristic.

Patient characteristic	Occurrence of VTE	
	Yes	No
Race – no. (%)		
Black	24 (32.4)	50 (67.6)
White/Caucasian	96 (21.5)	351 (78.5)
Other	3 (9.7)	28 (90.3)
Histology – no. (%)		
NSCLC	115 (23.7)	370 (76.3)
SCLC	8 (11.9)	59 (88.1)
First-line treatment type – no. (%)		
Chemotherapy	25 (24.5)	77 (75.5)
Chemotherapy + Immunotherapy	39 (20.4)	152 (79.6)
Immunotherapy	21 (16.8)	104 (83.2)
TKI	38 (28.4)	96 (71.6)
Oncogene Driver Mutation – no. (%)		
ALK	4 (20.0)	16 (80.0)
BRAF	5 (21.7)	18 (78.3)
EGFR	35 (28.2)	89 (71.8)
KRAS	29 (29.0)	71 (71.0)
MET	7 (38.9)	11 (61.1)
RET	1 (16.7)	5 (83.3)
ROS1	1 (12.5)	7 (87.5)
Baseline Khorana Score – no. (%)		
High	15 (20.3)	59 (79.7)
Low	108 (22.6)	370 (77.4)

VTE, venous thromboembolism; NSCLC, non-small cell lung cancer; SCLC, small cell lung cancer; TKI, tyrosine kinase inhibitor; ALK, anaplastic lymphoma kinase; BRAF, v-raf murine sarcoma viral oncogene homolog B1; EGFR, epidermal growth factor receptor; KRAS, kirsten rat sarcoma viral oncogene homolog; MET, mesenchymal epithelial transition factor receptor; RET, rearranged during transfection; ROS1, c-ros oncogene 1.

### 3.3 VTE incidence by khorana risk score

**Figure 3:**
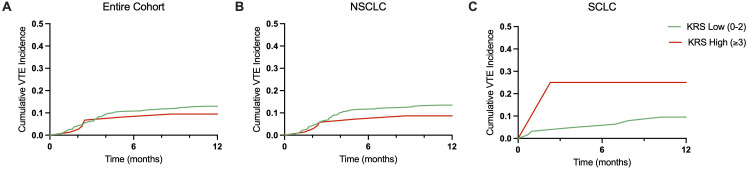
Cumulative VTE incidence over 12 months by Khorana Risk Score. **(A)** Cumulative VTE incidence for low (0-2, green line) and high (≥3, red line) KRS for the entire patient cohort over 12 months. **(B)** Cumulative VTE incidence for low and high KRS in NSCLC patients over 12 months. **(C)** Cumulative VTE incidence for low and high KRS in SCLC patients over 12 months.

Baseline KRSs were calculated for all 552 patients in our cohort: 478 patients (86.6%) had a low-risk KRS (score = 0-2), and 74 patients (13.4%) had a high-risk KRS (score ≥3). VTE events occurred in 108 patients with low-risk KRS (22.6%) and 15 patients with high-risk KRS (20.3%) during the entire follow-up period. No statistically significant difference in VTE incidence was observed based on KRS categorization (Pearson chi-square test, p=0.66). Among patients with low-risk KRS, cumulative VTE incidences (percent with event) were 10.7% at 6 months and 13.0% at 12 months (**Figure 3A**). Among patients with high-risk KRS, cumulative VTE incidences were 8.1% at 6 months and 9.5% at 12 months.

Similar trends were observed by histology sub-type: in NSCLC patients with low-risk KRS (n=415), cumulative VTE incidences were 11.6% at 6 months and 13.5% at 12 months (**Figure 3B**). Among NSCLC patients with high-risk KRS (n=69), cumulative VTE incidences were 7.2% at 6 months and 8.7% at 12 months. In SCLC patients with low-risk KRS (n=63), cumulative VTE incidences were 4.8% at 6 months and 9.5% at 12 months (**Figure 3C**). Among SCLC patients with high-risk KRS (n=4), cumulative VTE incidences were 25.0% at 6 months and 25.0% at 12 months.

### 3.4 VTE incidence by driver mutation

VTEs were observed in 82 patients harboring a driver mutation and 41 patients without a driver mutation (**Figure 2**). By driver mutation type, VTEs occurred in 4 out of 20 patients with *ALK* rearrangements, 5 out of 23 patients with *BRAF* mutations, 35 out of 124 patients with *EGFR* mutations, 29 out of 100 patients with *KRAS* mutations, 7 out of 18 patients with *MET* mutations, 1 out of 6 patients with *RET* rearrangements, and 1 out of 8 patients with *ROS1* rearrangements. Patients with a driver mutation had a statistically significant increased risk of VTE compared to patients that did not (Pearson chi-square test, p=0.002). No statistically significant increased risk of VTE was observed by individual oncogene driver mutation, although trends towards significance were seen in patients with *EGFR* mutations (Pearson chi-square test, p=0.08), *MET* mutations (Pearson chi-square test, p=0.09), and *KRAS* mutations (Pearson chi-square test, p=0.07).

### 3.5 Impact of anticoagulant and anti-platelet use on VTE incidence

Seventy-one patients (12.9%) in our cohort were prescribed and taking therapeutic anticoagulation for prior VTE events, atrial fibrillation, arterial thrombi, or another indication prior to their lung cancer diagnosis and treatment initiation. Of these patients, 4 patients developed VTE while on therapeutic anticoagulation, while 67 did not develop VTE. The use of therapeutic anticoagulation was associated with a statistically significant reduction in VTE incidence (Pearson chi-square test, p=0.001). Eleven patients with a high KRS were prescribed therapeutic anticoagulation prior to lung cancer diagnosis and initiation of treatment. Of these patients, 0 developed VTE while on therapeutic anticoagulation.

177 patients (32.1%) in our cohort were prescribed and taking an anti-platelet agent prior to lung cancer diagnosis and initiation of treatment. No statistically significant difference in VTE incidence was observed based on use of an anti-platelet agent; 35 patients developed VTE while receiving anti-platelet therapy, while 142 did not develop VTE (Pearson chi-square test, p=0.331).

### 3.6 Impact of VTE on overall survival

**Figure 4:**
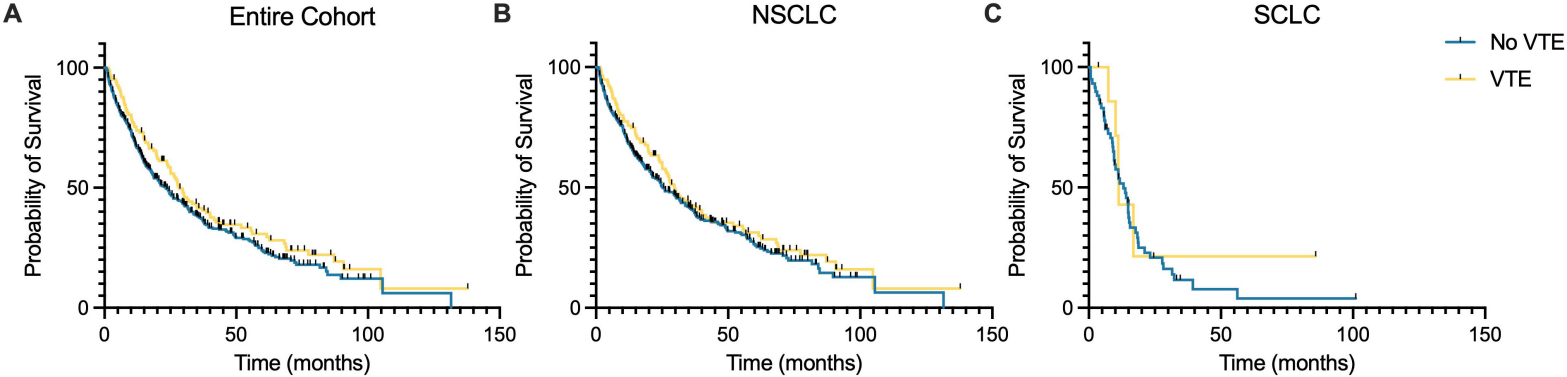
Kaplan-Meier curves of overall survival stratified by VTE status. Survival probability over time (months) is shown for patients with VTE (blue line) and without VTE (gold line) for the entire patient cohort **(A)**, those with NSCLC histology **(B)**, and those with SCLC histology **(C)**. No statistically significant difference in overall survival was observed when stratified by the occurrence of a VTE.

Landmark OS was calculated at the 3-, 6-, and 12-month time points for the entire cohort and for the NSCLC and SCLC histologic subgroups. Detailed data on OS are provided in **Table 4**. For the entire cohort, landmark OS was 21.1, 24.4, and 30.4 months at the 3-, 6-, and 12-month time points, respectively. Stratified by VTE status, landmark OS was 27.4, 28.8, and 34.8 months at the 3-, 6-, and 12-month time points, respectively, for those that experienced a VTE and 19.5, 22.8, and 29.8 months for those that did not. No statistically significant difference in OS was observed by VTE status for the entire cohort or by histologic subtype (**Figure 4**).

**Table 4:** Landmark overall survival stratified by presence or absence of VTE.

Group – Landmark time	No. (%)	Median OS (days, IQR)
Entire cohort – 3 months	496	643 (335-1208)
VTE +	117 (23.6)	843 (434-1466)
VTE -	379 (76.4)	592 (319-1143)
Entire cohort – 6 months	445	741 (422-1325)
VTE +	110 (24.7)	877 (464-1583)
VTE -	335 (75.3)	695 (410-1235)
Entire cohort – 12 months	353	925 (593-1643)
VTE +	91 (25.8)	1057 (713-1854)
VTE -	262 (74.2)	905 (561-1570)
NSCLC cohort – 3 months	435	723 (362-1324)
VTE +	109 (25.1)	862 (453-1527)
VTE -	326 (74.9)	644 (350-1252)
NSCLC cohort – 6 months	394	799 (445-1560)
VTE +	103 (26.1)	907 (538-1637)
VTE -	291 (73.9)	742 (427-1421)
NSCLC cohort – 12 months	324	966 (615-1704)
VTE +	88 (27.2)	1069 (719-1825)
VTE -	236 (72.8)	932 (593-1659)
SCLC cohort – 3 months	61	341 (222-559)
VTE +	8 (13.1)	338 (244-497)
VTE -	53 (86.9)	352 (215-564)
SCLC cohort – 6 months	51	443 (294-637)
VTE +	7 (13.7)	341 (304-510)
VTE -	44 (86.3)	445 (289-683)
SCLC cohort – 12 months	29	562 (452-961)
VTE +	3 (10.3)	510 (457-2588)
VTE -	26 (89.7)	564 (450-955)

Univariate and multivariate Cox proportional hazards regression was performed to evaluate the association between baseline clinical factors and OS, stratified by VTE status. In multivariate analysis, NSCLC histology was independently associated with a lower hazard of death (HR 0.56, 95% CI 0.41-0.76, p<0.001) when compared to SCLC histology. Similarly, the presence of an oncogenic driver mutation (HR 0.72, 95% CI 0.58-0.91, p=0.005) and Caucasian/White race (HR 0.67, 95% CI 0.51-0.88, p=0.004) were associated with improved OS. Baseline KRS and sex were not significantly associated with survival; see **Table 5** for full univariate and multivariate results.

**Table 5:** Univariate and multivariate cox regression for overall survival stratified by presence or absence of VTE.

Variable	Univariate HR (95% CI)	p-value	Multivariate HR (95% CI)	p-value
NSCLC vs. SCLC	0.50 (0.37-0.67)	<0.001	0.56 (0.41-0.76)	<0.001
AGA (Yes vs. No)	0.66 (0.53-0.82)	<0.001	0.72 (0.58-0.91)	0.005
High vs. Low KRS	1.20 (0.89-1.61)	0.226	1.29 (0.95-1.73)	0.099
Male vs. Female	0.94 (0.77-1.15)	0.559	1.00 (0.82-1.24)	0.972
White/Caucasian vs. Non-White	0.67 (0.51-0.87)	0.003	0.67 (0.51-0.88)	0.004

## 4 Discussion

In this real-world, retrospective analysis of patients with advanced lung cancer, the KRS did not effectively differentiate between patients at low and high risk for VTE, as we did not observe a significant difference in VTE incidence between patients with low and high baseline KRS. These results are consistent with previous reports. We observed higher rates of VTE in Black patients, patients with NSCLC histology, and patients harboring a driver mutation. We did not observe differences in VTE incidence based on initial treatment type, and the occurrence of a VTE did not impact OS for the entire cohort nor for the NSCLC and SCLC histologic subgroups.

Multiple previous retrospective studies have identified the limitations of the KRS in the context of lung cancer, similar to our findings (21, 22, 30). The KRS has also been evaluated prospectively in lung cancer patients in the noninterventional CANTARISK study, where it was not significantly associated with VTE in either univariate or multivariate analyses (10). Given our observed associations between NSCLC histology, the presence of a driver mutation, and increased risk of VTE, the limitations of the KRS may be because lung cancer specific risk factors were not incorporated into the score. Other clinical factors such as stage at diagnosis, disease burden, and the presence of visceral metastasis may impact VTE risk but are not integrated into the KRS (16, 27). The Caprini and Padua scores have also been studied as predictive models for assessing risk of VTE; however, these models have not been prospectively validated in patients with malignancy (31–33). Our findings highlight the limited utility of the KRS for VTE risk stratification in advanced lung cancer patients, particularly those with NSCLC, and the need for further risk stratification tools in this population. In addition to the parameters currently included in the KRS, lung cancer histology and the presence or absence of a driver mutation would be the most useful characteristics to validate and incorporate into future risk assessment models.

We observed higher rates of VTE in those patients with NSCLC compared to those with SCLC, similar to prior reports (17, 34). As shown in **Figure 3**, the majority of observed VTEs occurred during the first 3 months after initial lung cancer diagnosis, with a plateau in incidence in the subsequent months. If these findings are validated prospectively, future strategies incorporating prophylactic anticoagulation could consider shorter durations of anticoagulation in lung cancer patients based in part on this observation. Differing rates of VTE among the various histologic subtypes of lung cancer are thought to be due to multiple reasons. Among NSCLC patients, those with adenocarcinoma histology have been shown to be at an increased risk of VTE compared to those with squamous cell carcinoma histology (17, 35). Pre-clinical studies have shown that mucins produced by carcinomas can activate platelets through interactions with leukocyte l‐selectin and platelet P‐selectin, resulting in the generation of microthrombi and increased risk of VTE (36). Furthermore, we observed increased rates of VTE in Black patients compared to Caucasian and other patients, although our cohort included a small number of Black patients overall. This observation has been previously reported across multiple tumor types; however, the underlying reasons for this increased risk are currently unknown (37, 38).

The presence of a driver mutation in metastatic NSCLC has both prognostic and predictive significance. Patients in our study harboring a driver mutation had a statistically significant increase in VTE compared to those without a mutation. We did not observe a significant increase in VTE incidence when stratified by individual mutation type. However, a trend towards increased risk of VTE was observed in patients with *EGFR*, *MET*, and *KRAS* mutations. A meta-analysis by Qian et al. found that patients whose tumors harbor *ROS1* or *ALK* rearrangements had the highest incidence of VTE, while no statistically significant increase in risk of VTE was seen in those with *KRAS* or *EGFR* mutations (23). EGFR mutations have been shown to be negatively associated with risk of VTE in other analyses (25). Our results differ from these studies, as we observed a trend towards increased risk of in VTEs in patients with *EGFR* (p=0.08) and *KRAS* (p=0.09) mutations compared to wild-type patients. The impact of treatment modality on VTE incidence in *EGFR* mutation positive NSCLC is unknown; however, in the MARIPOSA trial comparing amivantamab and lazertinib to osimertinib, patients treated with the combination had a 37% risk of VTE compared to 9% in patients receiving osimertinib (39). Following this observed increase in VTE risk, the trial protocol was amended to include recommendations for prophylactic anticoagulation for the first 4 months (40). Our study did not include patients treated with this regimen, however, and thus this does not explain the increase in VTEs in patients with *EGFR* mutations in our cohort. Given the small number of patients in our cohort with *ROS1* and *ALK* rearrangements, we cannot draw conclusions about these patients and VTE risk.

Our study is limited by its single center, retrospective design. A survival bias may also exist, as patients with NSCLC and or a driver mutation may have longer OS and thus may have more time to develop VTE compared to those with SCLC or no observed driver mutation. Additionally, given low use of VTE primary prophylaxis in our cohort, we are unable to evaluate the impact of prophylactic anticoagulation on VTE risk in this population. Despite these limitations, our study adds valuable real-world insights into the patterns and predictors of VTE in lung cancer patients, with long-term, longitudinal follow-up that complements prior registry-based and prospective research.

In conclusion, our results suggest that incorporating patient, molecular, and histology-specific factors may improve risk prediction models for VTE in lung cancer. Currently, the KRS is the most widely used risk stratification tool for VTE in patients with cancer. A high KRS score suggests potential need for prophylactic anticoagulation, however as this study shows, application of the KRS to lung cancer patients may not be appropriate given our observation that a high KRS does not correlate with increased VTE incidence. Further studies to evaluate the risk of VTE in mutational subsets identified in our study are warranted, and it remains unclear whether these particular subsets could benefit from prophylactic anticoagulation. It is also unclear whether prophylactic anticoagulation in high-risk patients would have an impact on OS. Taken together, our findings support the need for revised or alternative VTE risk stratification tools tailored to patients with lung cancer. Prospective studies are warranted to validate our findings and to evaluate whether integrating certain clinical factors can guide prophylactic anticoagulation strategies more effectively than current models.

## Data Availability

The raw data supporting the conclusions of this article will be made available by the authors, without undue reservation.
